# Keratinocyte Growth Factor Combined with a Sodium Hyaluronate Gel Inhibits Postoperative Intra-Abdominal Adhesions

**DOI:** 10.3390/ijms17101611

**Published:** 2016-09-22

**Authors:** Guangbing Wei, Cancan Zhou, Guanghui Wang, Lin Fan, Kang Wang, Xuqi Li

**Affiliations:** 1 Department of General Surgery, the First Affiliated Hospital of Xi’an Jiaotong University, Xi’an 710061, Shaanxi, China; weiguangbing1208@163.com (G.W.); amon@mail.xjtu.edu.cn (G.W.); linnet@mail.xjtu.edu.cn (L.F.); wangkang5754@163.com (K.W.); 2 Department of Hepatobiliary Surgery, the First Affiliated Hospital of Xi’an Jiaotong University, Xi’an 710061, Shaanxi, China; trytofly@stu.xjtu.edu.cn

**Keywords:** peritoneum, mesothelial cells, regeneration, postoperative adhesions, keratinocyte growth factor

## Abstract

Postoperative intra-abdominal adhesion is a very common complication after abdominal surgery. One clinical problem that remains to be solved is to identify an ideal strategy to prevent abdominal adhesions. Keratinocyte growth factor (KGF) has been proven to improve the proliferation of mesothelial cells, which may enhance fibrinolytic activity to suppress postoperative adhesions. This study investigated whether the combined administration of KGF and a sodium hyaluronate (HA) gel can prevent intra-abdominal adhesions by improving the orderly repair of the peritoneal mesothelial cells. The possible prevention mechanism was also explored. The cecum wall and its opposite parietal peritoneum were abraded after laparotomy to induce intra-abdominal adhesion formation. Animals were randomly allocated to receive topical application of HA, KGF, KGF + HA, or normal saline (Control). On postoperative day 7, the adhesion score was assessed with a visual scoring system. Masson’s trichrome staining, picrosirius red staining and hydroxyproline assays were used to assess the magnitude of adhesion and tissue fibrosis. Cytokeratin, a marker of the mesothelial cells, was detected by immunohistochemistry. The levels of tissue plasminogen activator (tPA), interleukin-6 (IL-6), and transforming growth factor β1 (TGF-β1) in the abdominal fluid were determined using enzyme-linked immunosorbent assays (ELISAs). Western blotting was performed to examine the expression of the TGF-β1, fibrinogen and α-smooth muscle actin (α-SMA) proteins in the rat peritoneal adhesion tissue. The combined administration of KGF and HA significantly reduced intra-abdominal adhesion formation and fibrin deposition and improved the orderly repair of the peritoneal mesothelial cells in the rat model. Furthermore, the combined administration of KGF and HA significantly increased the tPA levels but reduced the levels of IL-6, tumor necrosis factor α (*T*NF-α) and TGF-β1 in the abdominal fluid. The expression levels of TGF-β1, fibrinogen and α-SMA protein and mRNA in the rat peritoneum or adhesion tissues were also down-regulated following the combined administration of KGF and HA. The combined administration of KGF and HA can significantly prevent postoperative intra-abdominal adhesion formation by maintaining the separation of the injured peritoneum and promoting mesothelial cell regeneration. The potential mechanism may be associated with rapid mesothelial cell repair in the injured peritoneum. This study suggests that combined administration of KGF and HA may be a promising pharmacotherapeutic strategy for preventing abdominal adhesions, which is worth further study, and has potential value in clinical applications.

## 1. Introduction

Postoperative intra-abdominal adhesions are a very common complication of abdominal surgery that may occur in 90% to 95% of patients undergoing abdominal surgery [1,2,3]. These unavoidable postoperative adhesions can cause a series of clinical problems, such as intestinal obstruction, postoperative abdominal and pelvic pain, female infertility, and difficult access in a subsequent surgery [4,5]. Ten percent of patients with intestinal obstructions caused by intra-abdominal adhesions must undergo adhesiolysis, for which the mortality rate is between 5% and 20%. The recurrent rate is very high after surgical treatment of adhesive intestinal obstructions. Without effective prevention of adhesions, the recurrence rate is 12% within 41 months after surgery; the recurrent risk is still considerable even 20 years after surgery. Studies of postoperative intra-abdominal adhesions have been ongoing since the emergence of surgery [6], but an effective medication that has a reliable effect and fewer side effects has not been identified. Clinically, many medications and approaches have been tested; however, the results have been inconclusive. Thus, a current clinical problem that remains to be solved is to identify an ideal strategy that prevents intra-abdominal adhesions [7].

Intra-abdominal adhesion formation results from fibrin exudation and deposition caused by a series of inflammatory processes in the injured peritoneum; furthermore, a decreased ability to dissolve fibrin at the injured sites can also lead to adhesion formation [8]. Peritoneal injury can induce the production of a fibrinogen-rich serous exudate, which activates the coagulation system and converts fibrinogen into fibrin to form deposits. Fibrin deposits can degrade into fibrin degradation products, and then, the injured peritoneum can be repaired by mesothelial cell regeneration. The degeneration of fibrosis is mainly completed by the peritoneal fibrinolytic system. Tissue plasminogen activator (tPA) and plasminogen activator inhibitor type 1 (PAI-1) of mesothelial cells, the main components of the peritoneal fibrinolytic system, play key roles in adhesion formation and development. Fibrin deposition can persist and form permanent fibrous adhesions when fibrinolytic activity is decreased due to poor mesothelial cell regeneration.

To this end, mesothelial injury is considered to be one of the causes of peritoneal adhesion formation [9]. The integrity of the peritoneal mesothelial cell layer is closely associated with fibrinolytic activity. Moreover, the loss of or poor proliferation and migration of mesothelial cells is another factor that promotes adhesion formation [10]. Peritoneal mesothelial cells not only form the smooth surface of the peritoneum but also exhibit fibrinolytic activity. Mesothelial cells are considered as the primary source of tPA in the serous cavity; their fibrinolytic activity in serous cavities plays a decisive role in local fibrin deposition and removal [10]. Normally, the balance between fibrin formation and dissolution of peritoneal mesothelial cells is maintained. However, when some pathological factors, such as peritoneal inflammation, mechanical damage, tissue ischemia, and foreign body implantation, are present, the mesothelial cells can be injured, reducing their fibrinolytic capacity. An imbalance between fibrin exudation and fibrinolysis can subsequently lead to tissue organization and adhesion formation.

The target of keratinocyte growth factor (KGF) is epithelial cells (including mesothelial cells). KGF can promote epithelial cell proliferation and growth and increase the rate of epithelialization [11,12]. KGF has rarely been reported to prevent intra-abdominal adhesions by promoting mesothelial cell regeneration. KGF has been shown to improve mesothelial cell proliferation, which may enhance fibrinolytic activity to potentially suppress postoperative adhesions [13]. This study investigated whether the combined administration of KGF and sodium hyaluronate (HA) can prevent intra-abdominal adhesions in rats by promoting the orderly repair of the injured peritoneal mesothelial cell layer. The possible prevention mechanisms were also explored.

## 2. Results

### 2.1. The Combined Administration of Keratinocyte Growth Factor (KGF) and Sodium Hyaluronate (HA) Significantly Reduced the Abdominal Adhesion Score in the Rat Model Using the Visual Scoring System

**Figure 1:**
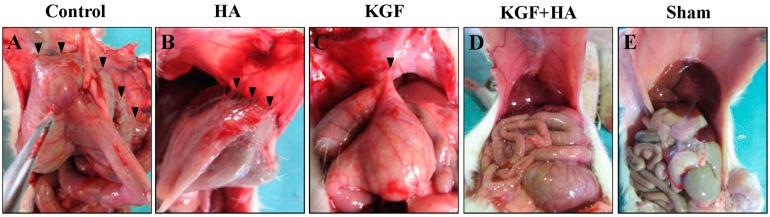
The combined administration of keratinocyte growth factor (KGF) and sodium hyaluronate (HA) prevented postoperative abdominal adhesion formation in rats. Representative images showing the intra-abdominal adhesions (black triangle) or the injured areas on the opposite parietal peritoneum on postoperative day 8 in each group of rats. (**A**) A large area of dense adhesions formed and the adhesions were difficult to separate in the control group; (**B**) Moderate adhesions were less severe in the HA group of rats than in the control group; (**C**) The relatively significant adhesions in the KGF group of rats were similar to those in the HA group in magnitude; (**D**) Very mild adhesions in some rats in the KGF plus HA group; (**E**) No adhesions were observed in the sham group. The surfaces of the parietal peritoneum and cecum were smooth.

Animal death was not observed, and all animals completed the entire experimental protocol. No wound disruption, wound infection or intra-abdominal infections were observed in any of the groups. The magnitude of abdominal adhesions between groups was different (Figure 1). Adhesion formation was rarely observed in the sham laparotomy group, and the surface of the parietal peritoneum was smooth. Sheet-like adhesions were observed in the control group, which were difficult to separate. In the HA group, the jelly-like HA gel on the animal’s cecal surface was completely absorbed, and the partially injured visceral surface was repaired. Strip-like adhesions were observed between the parietal peritoneum and cecum wound bed or between the omentums. The magnitude of adhesion information was milder than the control group. The magnitude of adhesion formation in the KGF group was similar to the HA group. In the animals of the KGF and HA groups, there was a low magnitude of adhesion formation, which appeared loose and had a thin thread-like morphology.

**Figure 2:**
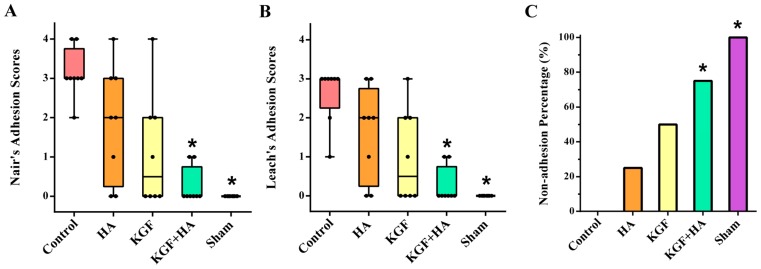
Comparison of intra-abdominal adhesion score in each group (*n* = 8). HA or KGF has a non-obvious trend to reduce adhesions in the rats. The magnitude of adhesion was significantly decreased in the KGF plus HA group. (**A**) Intra-abdominal adhesion score based on Nair’s classification (* *p* < 0.05, compared with the control group); (**B**) Intra-abdominal adhesion score based on Leach’s classification (* *p* < 0.05 compared with the control group); (**C**) Comparison of the non-adhesion rate (* *p* < 0.05, Fisher’s exact test).

The magnitude of intra-abdominal adhesions was scored according to Nair’s classification, and there was a significant difference among the five groups of rats (*p* < 0.05) (Figure 2A). Compared to the control group, HA or KGF reduced the postoperative adhesions in the rats respectively. The differences were not significant. In contrast, the magnitude of intra-abdominal adhesion formation was significantly decreased in the KGF plus HA group of rats (*p* < 0.05). In addition, the adhesion score was the lowest in the KGF plus HA group when using the scale described by Leach et al. (Figure 2B). The proportion of rats without abdominal adhesions in all groups was analyzed; we found that the rate of animals without adhesion formation was 75% in the KGF plus HA group and was significantly increased compared to the HA and KGF groups. The above-reported results indicated that the combined administration of KGF and HA can significantly prevent postoperative intra-abdominal adhesion formation in rats.

### 2.2. The Combined Administration of KGF and HA Decreased Collagen Deposition in the Injured Peritoneum of the Rat Model

**Figure 3:**
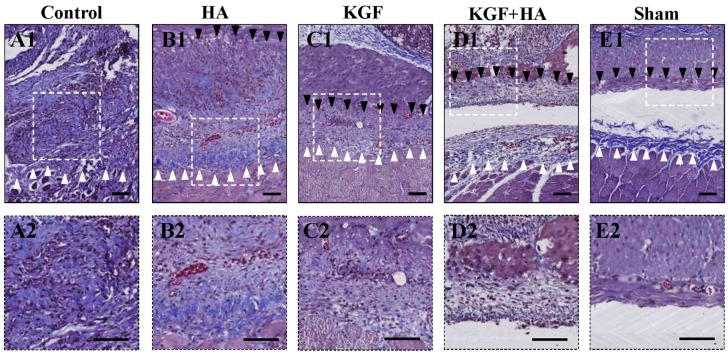
Masson’s trichrome-stained images showing the intra-abdominal adhesions (black triangle) or injured areas on the opposite parietal peritoneum (white triangle) in each group of rats. (100× magnification pictures in upper plate, 200× magnification pictures in below plate; the black scale bar represents 100 μm). (**A1**,**2**) Image showing the compact structure and significant hyperplasia of the collagen fibers (dark blue staining) in the control group; (**B1**,**2**) Image showing the collagen fibers (dark to light blue staining) in the HA group, in which the staining is somewhat lighter than the control group; (**C1**,**2**) The abundance of collagen fiber hyperplasia in the KGF group was similar to the HA group; (**D1**,**2**) The collagen fiber was significantly impoverished in the KGF plus HA group; (**E1**,**2**) The structure of the collagen fibers was clear and intact with a very loose arrangement in the control group.

**Figure 4:**
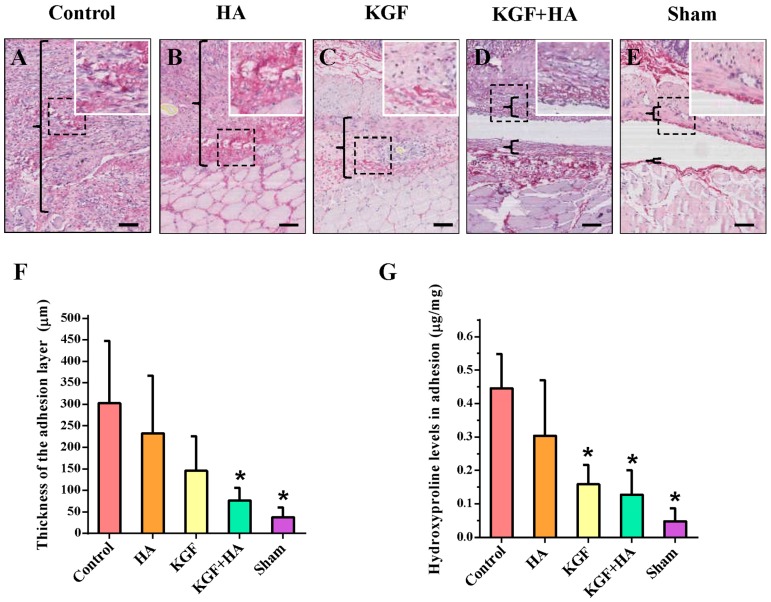
The magnitude of fibrosis in the intra-abdominal adhesions or the injured areas on the opposite parietal peritoneum in each group of rats (*n* = 8). (**A**–**E**) Representative images of picrosirius red staining (100×; insets, 200×) in each group. The adhesive tissues are located between the black brackets. The black scale bar represents 100 μm; (**F**) The thickness of the collagen deposits in the adhesive tissue of each group of rats (* *p* < 0.05 compared with the control group); (**G**) Hydroxyproline content in the adhesion tissues of each group of rats (* *p* < 0.05 compared with the control group).

Collagen fibers are stained blue by Masson’s trichrome staining (Figure 3). The structure of the collagen fibers was intact, and collagen fiber proliferation was not observed in the sham operation group, whereas collagen fibers were stained dark blue in the control group, representing obvious hyperplasia. In the HA and KGF groups, collagen fibers were stained different shades of blue, which was somewhat lighter than the staining in the control group. More importantly, the area size and density of collagen fibers were not only significantly reduced in the KGF plus HA group compared with the control group but also significantly reduced compared with the HA and KGF groups. In addition, we examined the thickness of the abdominal adhesions between the injured rat peritoneum using picrosirius red staining of collagen fibers (Figure 4A). Compared with the HA and KGF groups, the collagen fibers in the rat adhesion tissue appear more loose, and the thickness of the abdominal adhesions was significantly decreased in the KGF plus HA group (*p* < 0.05) (Figure 4B). Furthermore, the combined administration significantly reduced the hydroxyproline content of the adhesion tissue in the animals of the KGF plus HA group (*p* < 0.05) (Figure 4C). Thus, our data suggested that the combined administration of KGF plus HA could decrease collagen deposition during adhesion formation in the rat model.

### 2.3. The Combined Administration of KGF and HA Can Reduce Inflammatory Infiltration in the Injured Rat Peritoneum

**Figure 5:**
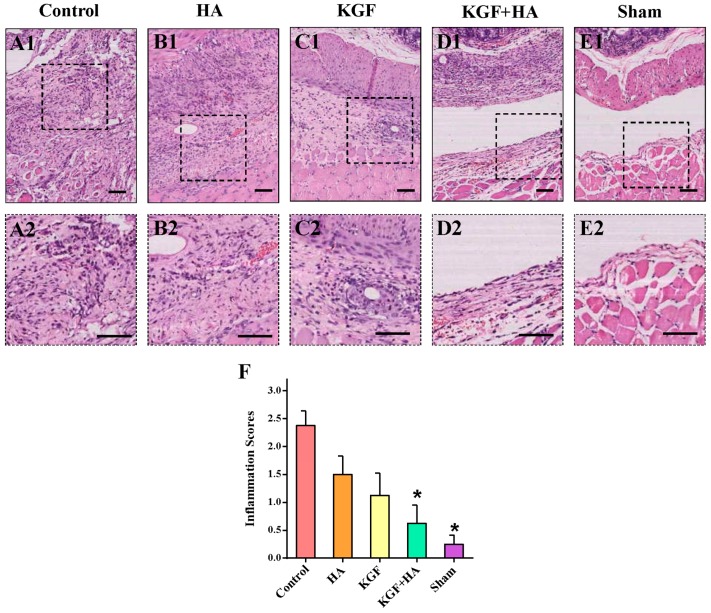
Inflammatory infiltration in the intra-abdominal adhesions or the injured areas on the opposite parietal peritoneum in each group of rats (*n* = 8). (**A1**,**2**–**E1**,**2**) Representative images of HE staining in the intra-abdominal adhesions or the injured areas on the opposite parietal peritoneum in each group of rats (100× magnification pictures in upper plate, 200× magnification pictures in below plate; the black scale bar represents 100 μm); (**F**) Inflammatory infiltration score for the adhesive tissue in each group of rats (* *p* < 0.05 compared with the control group).

We utilized the hematoxylin and eosin (HE) staining technique to compare the magnitude of inflammatory cell infiltration in the peritoneum (Figure 5A–E). The criteria for the inflammation score were listed in Table S3. In the control group, the accumulation of a number of inflammatory cells and some small abscesses were noted in the peritoneal tissue. The same inflammatory cells were also noted in the HA and KGF groups; however, the magnitude of the accumulation was less severe than that in the control group. Only a few scattered inflammatory cells were noted in the KGF plus HA group, indicating a significant decrease in inflammatory cell accumulation compared to that in the HA or KGF group. The inflammation scores were significantly different among the five groups of rats (Figure 5F), in which the highest score was observed in the control group. The score in the KGF plus HA group was not only significantly reduced compared with the control group (*p* < 0.05) but also significantly reduced compared with the HA and KGF groups (*p* < 0.05). Thus, the results have shown that the combined administration of KGF and HA significantly inhibited the accumulation of inflammatory cells in the peritoneum.

### 2.4. The Combined Administration of KGF and HA Promotes Mesothelial Cell Repair on the Injured Peritoneal Surface

**Figure 6:**
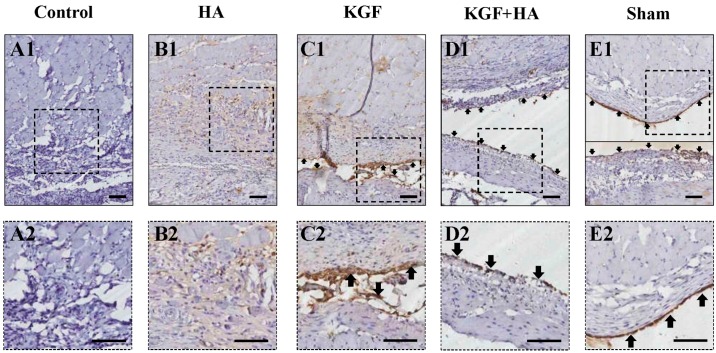
Immunohistochemical staining of cytokeratin in the intra-abdominal adhesions or the injured areas on the opposite parietal peritoneum in each group of rats (100× magnification pictures in upper plate, 200× magnification pictures in below plate; the black scale bar represents 100 μm). (**A1**,**2**) In the control group, the brown thin continuous line was completely interrupted; the interrupted brown line indicated the injured mesothelial cell layer; (**B1**,**2**, **C1**,**2**) The mesothelial cell layer (brown thin continuous line) was interrupted in the KGF and HA groups; (**D1**,**2**) In the KGF plus HA group, continuous brown lines were observed, indicating that the peritoneal mesothelial cell layer was completely repaired by regeneration; (**E1**,**2**) In the sham operation group, the parietal peritoneum and visceral peritoneum of the intestine surface were stained brown. A brown thin continuous line was formed, indicating that the peritoneal mesothelial cell layer was intact. Arrow: peritoneal mesothelial cell.

Immunohistochemical staining for cytokeratin (CK), a marker of mesothelial cells, was used to assess the continuity of the mesothelial cell layer of the injured rat peritoneum (Figure 6). The shape of representative mesothelial cells resembles cobblestones, and the cytokeratin staining is positive [14]. Our studies have observed the presence of a continuous and intact cell layer on the surface of the parietal and visceral peritoneum in the sham group. In contrast, mesothelial cells were not present on the surface of the injured parietal and visceral peritoneum in the control group. Similarly, mesothelial cell repair was not evident in the HA group. Mesothelial cell regeneration was noted in the KGF or KGF plus HA group, but, more importantly, a well-repaired mesothelial cell layer was noted in the KGF plus HA group, and its continuity was similar to the sham group. Therefore, the combined administration KGF and HA can significantly contribute to mesothelial cell repair on the surface of the injured peritoneum.

### 2.5. The Combined Administration of KGF and HA Inhibited the Severity of the Fibrous Changes in the Injured Peritoneum and/or Adhesion Tissue in the Rat Model

**Figure 7:**
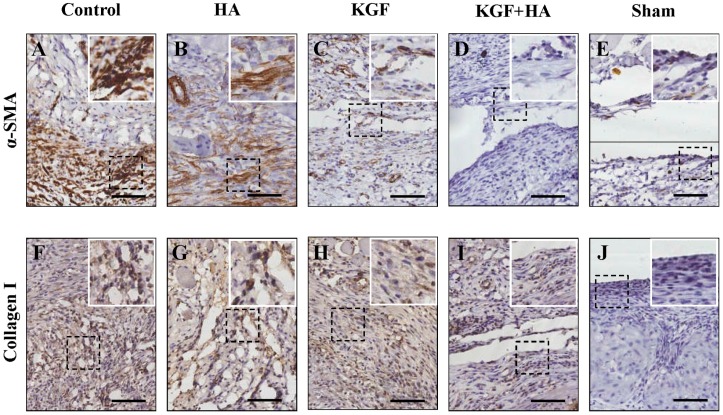
Immunohistochemical staining of α-smooth muscle actin (α-SMA), vimentin and collagen I in the intra-abdominal adhesions or the injured areas on the opposite parietal peritoneum in each group of rats (200×; insets, 400×). The black scale bar represents 100 μm. (**A**–**E**) α-SMA staining; (**F**–**J**) Collagen I staining.

Using immunohistochemical staining for α-smooth muscle actin (α-SMA), an activated fibroblast marker, the degree of fibrous changes in the injured rat peritoneum and/or adhesion tissue was examined (Figure 7A–E). In the sham laparotomy group, the peritoneum was intact, and no positive staining was observed. In the control group, a large amount of fusiform fibroblasts with positive brown staining were observed in the thick adhesive tissue. In the HA and KGF groups, the number of fibroblasts with positive α-SMA expression was slightly less than the number in the control group. However, in the KGF plus HA group, the degree of fibrous changes in the injured peritoneum and/or adhesion tissue was significantly reduced. Immunohistochemical staining for collagen I deposition (Figure 7F–J), a marker of fibrosis, showed that the amount of collagen I deposition on a scale from high to low was the control group, the HA group, the KGF group, the KGF plus HA group and the sham operation group. Thus, the results showed that the combined administration of KGF and HA could effectively inhibit fibrosis of the injured or adhesive peritoneum in a rat model.

### 2.6. The Combined Administration of KGF and HA Suppressed the Abdominal Fluid Levels of Tissue Plasminogen Activator (tPA) and the Pro-Inflammatory Cytokines Transforming Growth Factor β1 (TGF-β1) and Interleukin 1β (IL-1β) in the Rat Model

**Figure 8:**
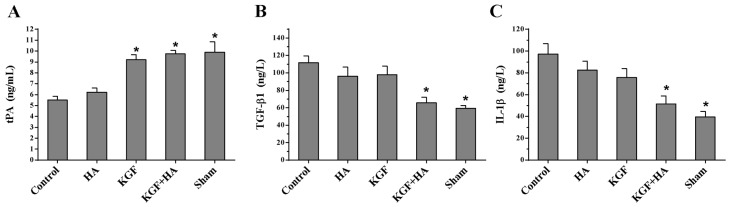
Abdominal fluid levels of tissue plasminogen activator (tPA) (**A**); transforming growth factor β1 (TGF-β1) (**B**); and interleukin 1β (IL-1β) (**C**) in each group (*n* = 8) (* *p* < 0.05 compared with the control group).

On postoperative day 7, the abdominal fluid levels of tPA, TGF-β1 and IL-1β were measured by ELISAs (Figure 8). The results revealed that the tPA levels in the abdominal fluid were significantly reduced in the control group compared with the sham operation group, but the levels of the pro-inflammatory cytokines TGF-β1 and IL-1β were significantly increased compared with the sham operation group, suggesting that surgical injury-induced adhesion formation resulted in decreased local fibrinolytic activity and significant inflammatory responses. Moreover, the KGF plus HA intervention could significantly inhibit the decrease in the tPA levels and the increase in the TGF-β1 and IL-1β levels (*p* < 0.05). This inhibitory effect was not significantly different in the HA group and the KGF group (*p* > 0.05). These results suggested that the combined administration inhibited the down-regulation of fibrinolytic activity and the significant release of inflammatory factors induced by the injured peritoneum during adhesion formation.

### 2.7. The Combined KGF and HA Treatment Inhibited Src Phosphorylation and Expression of TGF-β1, Fibrinogen, and α-Smooth Muscle Actin (α-SMA) in the Injured Peritoneum and/or Adhesion Tissue in the Rat Model

**Figure 9:**
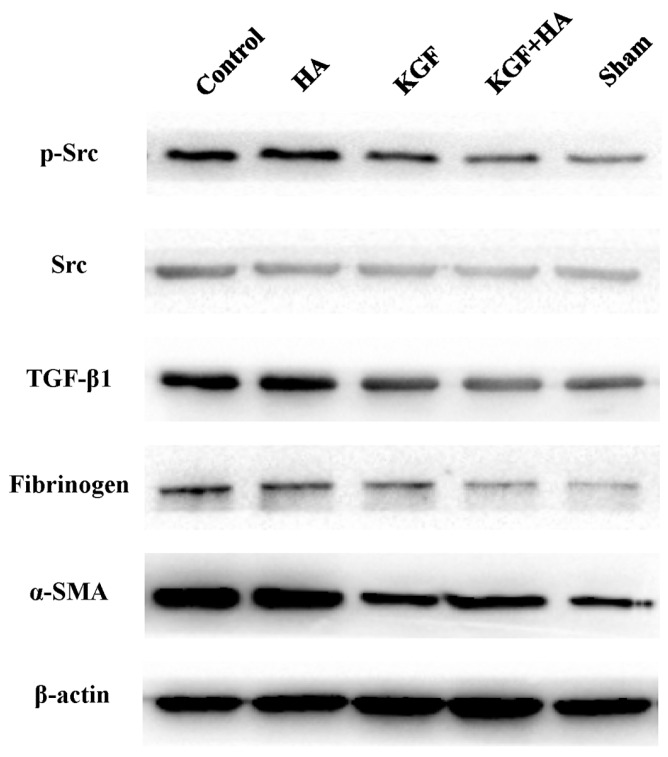
Expression of phosphorylated Src, Src, TGF-βl, fibrinogen and α-SMA protein in the intra-abdominal adhesions or the injured areas on the opposite parietal peritoneum by Western blotting.

The Western blot analysis showed that Src phosphorylation was significantly increased on postoperative day 7 in the KGF group and KGF plus HA group, suggesting that KGF can promote Src phosphorylation and activation (Figure 9). In addition, TGF-β1 expression in the abdominal adhesion tissue was significantly increased in the control group compared with the sham operation group. Compared with the control group, the HA and KGF groups did not show apparent TGF-β1 down-regulation. However, in the KGF plus HA group, expression of the TGF-β1 protein in the injured peritoneum and/or adhesion tissue was significantly decreased compared with that in the control, HA, and KGF groups (Figure 9). Moreover, we examined the expression of the fibrinogen and α-SMA proteins. With a similar pattern as the TGF-β1 levels in the groups, the expression levels of fibrinogen and α-SMA protein were reduced by the combined administration of KGF plus HA in the injured peritoneum and/or adhesion tissue (Figure 9).

**Figure 10:**
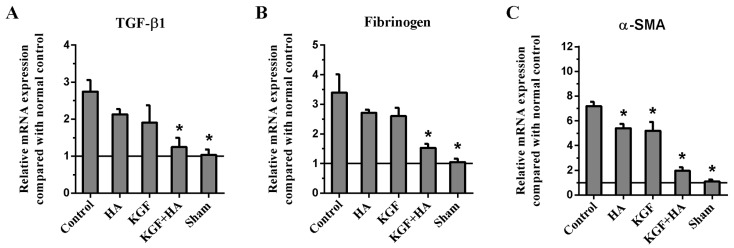
Expression of TGF-βl (**A**); fibrinogen (**B**) and α-SMA (**C**) mRNA in the intra-abdominal adhesions or the injured areas on the opposite parietal peritoneum by real-time RT-PCR (*n* = 8). (* *p* < 0.05 compared with the control group)

The expression levels of TGF-β1, fibrinogen and α-SMA mRNA in the injured peritoneum and/or adhesion tissue on postoperative day 8 were determined using the real-time RT-PCR technique (Figure 10). We found that the expression levels of TGF-β1, fibrinogen and α-SMA mRNA in the injured peritoneum and/or adhesion tissue were the lowest in the KGF plus HA group (*p* < 0.05). The mRNA expression levels were consistent with the protein expression levels shown in the Western blot analysis.

## 3. Discussion

The results of this study have shown that, compared to the administration of either KGF or HA alone, the combined administration of KGF and HA can more effectively prevent intra-abdominal adhesion formation in a rat model. This good preventative effect may be associated with the synergistic effect of the two agents, which can promote the repair of the mesothelial cell layer. Therefore, this study showed that the combined administration of KGF plus HA is a promising initial strategy for the prevention of intra-abdominal adhesions. It is worth continuing the study of its mechanism and translating the results into clinical practice.

At present, the following pharmacological preventive strategies against postoperative intra-abdominal adhesions should be considered [15,16,17]: (1) minimizing the initial inflammatory reaction and exudate; (2) inhibiting agglutination of the exudate; (3) promoting fibrinolysis; (4) using a physical barrier that can isolate the injured surface coated with fibrin; and (5) inhibiting fibroblast proliferation. Unfortunately, most of the regimens that have been used to prevent adhesion formation have limited effectiveness and considerable side effects [18]. It has been reported that intra-abdominal perfusion of crystalloid solutions and dextran injections are ineffective at preventing adhesions; moreover, the use of anti-inflammatory drugs has increased the risk of gastrointestinal anastomotic leakage. Currently, the most widely accepted method for the prevention of intra-abdominal adhesions is to place a medication barrier or gel between the injured peritoneal surfaces for at least 5 to 7 days. Some animal and clinical trials have shown that HA, chitosan and polyethylene glycol/polylactic acid films can indeed reduce adhesions [1,2,3], but others reported that the effectiveness of these medications is not significant [19]. The reason that the above-mentioned medications are not ideally effective in adhesion prevention may be because researchers have overlooked the function of mesothelial cells on the surface of the peritoneum.

Mesothelial cells are specialized epithelial cells that line the internal organs and body wall in the peritoneal, pleural, and pericardial cavities [20,21]. The surface of the serous peritoneum is an intact mesothelial cell layer. Intra-abdominal adhesions form between the injured surfaces of the two mesothelial cell layers [22]. Surgical injury may cause local tissue ischemia and hypoxia in the peritoneum [23], which trigger the inflammatory responses involving polymorphonuclear leukocytes, macrophages, fibroblasts and neovascularization and result in fibrin deposition for the initial adhesion formation [24,25]. Thereafter, the activation of the fibrinolytic system can degrade the fibrin deposits in the abdominal cavity. If fibrinolytic activation did not occur, fibroblasts would produce large amounts of collagen to form adhesions in 5 to 6 days after the injury of peritoneal mesothelial cell layer. Then, the mesothelial cells would completely cover the injured surface and adhesion area by proliferating to form fibrous adhesions, leading to permanent adhesion formation [26].

Mature adhesions can be effectively prevented if the intended techniques are used to enhance the regeneration ability of mesothelial cells and to rebuild the intact mesothelial cell layer in early intra-abdominal adhesions [27]. However, strategies to protect mesothelial cell regeneration are rarely reported, even though there have been a number of approaches described for the prevention of intra-abdominal adhesions. Bertram et al. [28] reported that intraperitoneal transplantation of isologous mesothelial cells resulted in a significant reduction of adhesion formation. Guo and colleagues [29] showed that high expression of sphingosine kinase (SPK1) can enhance both the proliferation and migration of mesothelial cells to accentuate the repair of mesothelial cells; SPK1 can also induce the cells to secrete plasminogen activator to enhance the peritoneal fibrinolytic capacity and ultimately prevent adhesion formation. These results suggest that the rapid enhancement of mesothelial cell regeneration is a possible strategy to prevent adhesion formation.

KGF is a single-chain polypeptide that was first isolated from the culture medium of human embryonic lung fibroblasts by Finch and Rubin in 1989 [30]. Its molecular weight is 26 to 28 kDa. The gene sequence analysis indicates that KGF is a member of the fibroblast growth factor (FGF) family and thus is known as FGF-7. KGF targets epithelial cells (including mesothelial cells) and may have potential mitogen activity and promotes proliferation in a variety of epithelial cells [31]. KGF can prevent and repair epithelium injuries of the skin, cornea, bladder, lungs, intestines, and liver through multiple approaches and multiple levels [32]. Lopes et al. [13] employed KGF to prevent swine pericardial adhesions in their study and suggested that the use of growth factors targeting mesothelial cell proliferation or regeneration can reduce the severity of adhesions. However, it has not been reported that KGF can prevent peritoneal adhesions. This study assessed the effect of KGF on adhesion formation in rats. The results have shown that KGF indeed reduced the severity of peritoneal adhesions and that the administration of KGF alone had approximately the same effect as the HA gel.

KGF function depends on the phosphorylation of the tyrosine kinase Src. Src can be phosphorylated by the KGF receptor (KGFR) after KGF binds KGFR [33] and thus exert its biological effects on downstream targets. In our study, we evaluated Src phosphorylation levels after the administration of KGF. We found that the levels of the phosphorylated Src protein were up-regulated in the peritoneal tissue after the administration of KGF to rats. This result suggests that KGF indeed plays a role in the local repair of the injured peritoneum.

KGFR is only distributed in epithelial cells (including mesothelial cells), and thus, KGF has no effect on fibroblasts and endothelial cells [11]. Therefore, the use of KGF can reduce adhesions without stimulating fibroblast proliferation and activation and thus can avoid aggravating adhesion fibrosis. Our experimental results showed that the degrees of collagen deposition and fibrosis in the adhesive tissue were reduced in the rats of the KGF intervention group compared with the control group.

Although KGF reduced the peritoneal adhesions, its effect was similar to the effect of the HA gel in clinical practice. The proportion of rats without adhesion formation did not reach 50% of the total number of rats. This result indicated that a considerable number of rats did not obtain a benefit from the KGF intervention. The possible reason is that the timing and speed of mesothelial cell repair is crucial for peritoneal adhesion prevention. Typically, mesothelial cell repair begins at 1 or 2 days and is completed in approximately 8–10 days after peritoneal injury [34] because peritoneal injury causes a large amount of fibrin deposition in the injured sites during this period. If the regenerated mesothelial cells cover the surface of the fibers and collagen, they can instead promote adhesion maturation and lead to permanent adhesion formation. Therefore, we suspect that the inhibition of fibrin deposition on the injured peritoneal surface during KGF-promoted mesothelial cell repair can greatly increase the effect on adhesion prevention.

HA gels are a commonly used barrier-like material for the clinical prevention of postoperative abdominal adhesions [1]. Hyaluronic acid is an acidic mucopolysaccharide. It is a normal glycosaminoglycan that is distributed in the extracellular matrix of the connective tissue in the human body. At physiological concentrations, hyaluronic acid molecules are entangled and form a disordered network of fibers that comprise its unique three-dimensional structure. It is difficult for some macromolecules, such as fibrinogen, collagen and proteoglycan, to enter this network structure. Hyaluronic acid enters the abdominal cavity in a colloidal state to form a jelly-like substance and covers the surface of the injured peritoneum to avoid the direct and effective contact between the surfaces. HA is a form of the sodium salt of hyaluronic acid, a naturally degraded and absorbable biomedical material [35]. Furthermore, HA can reduce the exudation of inflammatory cells, reduce the deposition of collagen at injured sites, and promote the physiological repair of injured tissue [36]. HA definitely plays a beneficial role in isolating the injured surface. This study showed that the barrier effect of HA also mildly or moderately reduced the magnitude of abdominal adhesions.

**Figure 11:**
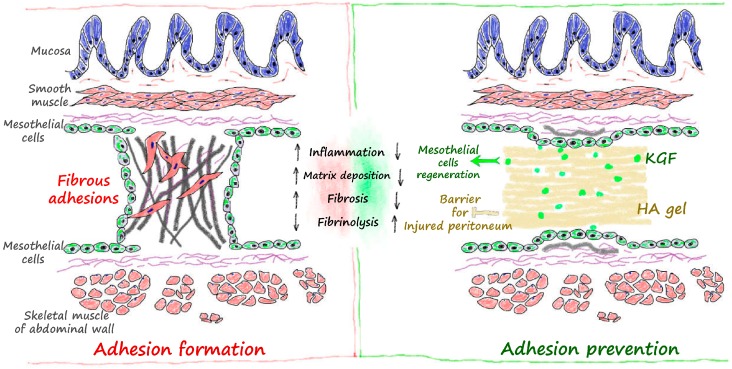
Mechanism by which the combined administration of KGF and HA prevents intra-abdominal adhesions. The HA gel can maintain the separation of the parietal and visceral peritoneum; KGF accentuates the regeneration and repair of the peritoneal mesothelial cell layer and thus the orderly repair of the integrity of the injured peritoneal mesothelial cell layer.

In theory, better prevention of adhesion formation can be achieved if a barrier material is used to maintain the separation between the parietal and visceral peritoneum before KGF accentuates the restoration of the integrity of the mesothelial cell layer of the peritoneum. The mechanisms by which KGF and HA prevent adhesions are different. Thus, we speculated that, theoretically, the combined administration of the two agents may be more efficacious in preventing adhesions. Our study also showed that the combined administration of KGF and HA can achieve better results in terms of adhesion severity and reducing the inflammatory cytokine levels compared to each treatment alone. The combined administration of KGF and HA significantly reduced the magnitude of inflammatory infiltration and collagen deposition at the injured peritoneal adhesions in rats. The combined administration can take advantage of the synergistic effects to achieve better results in the prevention of peritoneal adhesions (Figure 11).

There are some limitations and concerns with this study. KGF should be used with caution to prevent postoperative abdominal adhesions in the setting of abdominal tumor surgery because experimental studies suggested that KGF, a growth factor, may have the potential to promote tumor growth [36] or increase tumor resistance to chemotherapy [37]. Oelmann et al. [38] have proven that under the effects of recombinant KGF (r-KGF), the majority of tumor cell lines did not exhibit meaningful proliferation in vitro among 35 cancer cell lines of epithelial origin and 22 lymphoma and leukemia cell lines. Only five cancer cell lines (two lung cancer, one gastric cancer, one colon cancer and one breast cancer) exhibited statistically significant proliferation in a dose-dependent manner. Although KGF has been used at non-tumor sites to treat disease [39], people are still concerned about whether its use will cause tumor growth. Another issue of this present study regarded the lasting effective period of KGF in the body. KGF has poor stability and a short biological half-life; the biological activity of KGF is highly susceptible to environmental changes. Mesothelial cells require 8 days to repair the injured surface; however, the activity of intraperitoneally administered KGF cannot last for a long time due to the impact of a variety of biological enzymes. Thus, further improvements in the KGF dosage form are needed to maintain stable pharmacological effects on the body.

In summary, the present study has shown that the combined administration of KGF and HA can effectively prevent postoperative intra-abdominal adhesion formation in a rat model by maintaining the separation of the injured peritoneum and promoting mesothelial cell regeneration. The combined administration of KGF and HA has shown considerable advantages from the aspects of the inhibition of collagen deposition, tissue fibrosis, and inflammation. This study suggests that the combined administration of KGF and HA is a promising pharmacotherapeutic strategy in intra-abdominal adhesion prevention and also provides more ideas for the development of novel drugs to prevent adhesion formation.

## 4. Materials and Methods

### 4.1. Agents

The r-KGF was purchased from Prospec (Rehovot, Israel; CAT#: cyt-219) in a highly purified state (95%). Stock solutions were made after an initial dilution with sterile phosphate-buffered saline and stored at −20 °C at a 1000 ng/mL concentration. At the time of use, the stock solutions were thawed and diluted in sterile water. The sample for the KGF group consisted of a 15 mL solution containing 25 ng/mL r-KGF. The medical HA gel (10 mg/mL) was produced by Hangzhou Singclean Medical Products Co., Ltd., China. KGF at a 1000 ng/mL concentration was thawed and dissolved in the sterile hyaluronate gel. The final sample of KGF + HA gel contains 25 ng/mL r-KGF.

### 4.2. Surgical Procedures

Male Sprague-Dawley rats weighing 200 to 250 g were purchased from the Experimental Animal Center of Xi'an Jiaotong University. These animals were housed at room temperature (22 ± 2 °C), with free access to water and standard rat chow. All animal experiment protocols were approved by Xi'an Jiaotong University Experimental Animal Ethics Committee (No. 2015-156, 6 March 2015). The animals were anesthetized through the inhalation of methoxyflurane. The abdominal skin was prepared and disinfected with the povidone-iodine prior to the procedure. As previously described in the literature [40], a 2- to 3-cm-long lower abdominal midline incision was used to access the abdominal cavity. The pouch-like cecum was located in the right iliac fossa. The cecum wall and its opposite parietal peritoneum were abraded with sterile gauze until spot bleeding was observed. The abraded area was approximately 2–3 cm^2^ and was exposed to air for approximately 5 min. The bowels were arranged to ensure that the abraded cecum wall was opposite the abraded peritoneum. In the group with a sham operation, the abrasion and exposure was not performed. In the Control, HA, KGF or KGF + HA groups, a 1 mL normal saline, HA gel, r-KGF or HA gel containing r-KGF was applied to the abraded peritoneum and its surrounding areas, respectively. Interrupted 3-0 Vicryl sutures were used to close the peritoneum, the abdominal muscles, and the skin in 2 layers.

### 4.3. Adhesion Grade and Assessment

On day 8 after surgery, all rats were anesthetized, and an inverted “U” shape incision was used to open the abdomen. The magnitude of the intra-abdominal adhesions was assessed according to the adhesion grade criteria reported by Nair et al. [41] (Table S1) or Leach et al. [42] (Table S2). The investigators who assessed the adhesion grade were independent researchers and were blinded to the protocol. The rats were sacrificed after the assessment, and the specimens were collected for the subsequent studies.

### 4.4. Hematoxylin and Eosin (HE) Staining and Microscopic Histological Grading of Inflammation

The injured peritoneum and adhesion tissues were excised. Specimen fixation and section preparation were carried out. Then, HE staining was performed. The tissues were evaluated under a microscope in regards to the severity of inflammatory cell reaction by using the classification described by Mahdy et al. [43]. The standard was as follows: degree of inflammation (grade 0: absent or normal in number; grade 1: mild increase giant cells, occasional scattered lymphocytes and plasma cells; grade 2: moderate infiltration, giant cells with increased numbers of admixed lymphocytes, plasma cells, eosinophils, neutrophils; grade 3: massive infiltration, many admixed inflammatory cells, microabscesses present).

### 4.5. Picrosirius Red Staining for Collagen

Picrosirius red staining for collagen was achieved using 0.1% picrosirius red (Direct Red 80; Sigma-Aldrich, St. Louis, MO, USA) and counterstained with Weigert’s hematoxylin. The percentage of the positively stained area in eight randomly selected fields was evaluated using ImagePro Plus 5.0 software (Leica Qwin. Plus, Leica Microsystem Imaging Solutions Ltd., Cambridge, UK), and the average of the eight values was taken as the collagen content in the adhesions.

### 4.6. Immunohistochemistry

Immunohistochemical staining was performed using the SABC kit (Maxim, Fuzhou, China), according to the manufacturer’s instructions. The tissue sections were incubated with primary antibodies for cytokeratin AE1/AE3 (1:400 dilution), alpha smooth muscle actin (α-SMA) (1:500 dilution), and collagen I (1:300 dilution) overnight at 4 °C and incubated with the appropriate biotinylated secondary antibody for 30 min at room temperature, followed by a 30 min incubation with streptavidin peroxidase (Dako LSAB + HRP kit). After rinsing, the results were visualized using Diaminobenzidine tetrahydrochloride (DAB), and the slides were counterstained with hematoxylin.

### 4.7. Western Blot

Total proteins were extracted from the tissues using RIPA lysis buffer as previously described [44]. Cell lysates were resolved on 10% sodium dodecyl sulfate polyacrylamide gels and transferred to PVDF membranes. The membranes were blocked with 5% skim milk and incubated with primary antibodies overnight at 4 °C, which included an anti-phospho Src antibody (ab185617, Abcam, Cambridge, UK, 1:1000 dilution), anti-Src antibody (ab47405, Abcam, 1:600 dilution), anti-TGF-β1 antibody (sc-146, Santa Cruz Biotechnology, Dallas, TX, USA, 1:400 dilution), anti-fibrinogen antibody (sc-18029, Santa Cruz Biotechnology, 1:800 dilution), anti-α-SMA antibody (sc-53015, Santa Cruz Biotechnology, 1:800 dilution), and anti-β-actin antibody (sc-47778, Santa Cruz Biotechnology, 1:1000 dilution). The membranes were washed and incubated with the secondary antibodies for 2 h at room temperature. Protein expression was detected using a chemiluminescence system (Millipore, Billerica, MA, USA) according to the manufacturer’s specifications.

### 4.8. Real-Time RT-PCR

Real-time RT-PCR was performed to determine the messenger RNA (mRNA) levels of TGF-β1, fibrinogen, α-SMA and GAPDH. Total RNA was extracted using TRIzol reagent (Invitrogen, Carlsbad, CA, USA), and reverse transcription was performed using a PrimeScript RT reagent Kit (TaKaRa, Dalian, China). The real-time experiments were conducted on an iQ5 Multicolor Real-Time PCR Detection System (Bio-Rad, Hercules, CA, USA) using a SYBR Green Real-time PCR Master Mix (TaKaRa). The PCR reactions consisted of 5 s at 94 °C followed by 40 cycles at 94 °C for 30 s, 60 °C for 30 s, and 72 °C for 30 s. The PCR primer sequences are listed in Table S4. The comparative C(T) method was used to quantitate the expression of each target gene using GAPDH as the normalization control [45].

### 4.9. ELISA Quantification of Abdominal Fluid Levels of IL-6, Tumor Necrosis Factor *α* (TNF-α), TGF-β1

Abdominal fluid samples were collected from the abdomens of the animals and centrifuged at 3000 rpm for 30 min. The supernatant was stored at −20 °C. The concentrations of IL-6, tumor necrosis factor α (*T*NF-α), and TGF-β1 were measured using ELISA kits (R & D Systems, Minneapolis, MN, USA) according to the manufacturer’s instructions.

### 4.10. Hydroxyproline Determination

Hydroxyproline content was determined using a Hydroxyproline Assay Kit (Sigma-Aldrich) according to the manufacturer’s instructions. The tissue hydroxyproline levels, which were used as an indicator of the adhesion severity, are presented as micrograms of hydroxyproline per gram of protein.

### 4.11. Statistical Analyses

Categorical variables are listed as medians (minimum to maximum), and continuous variables are listed as averages and standard deviations. The categorical variables were evaluated by the Kruskal-Wallis H variance analysis for independent samples and the post hoc Mann-Whitney U test for multiple matches. Continuous variables were analyzed using one-way analysis of variance followed by the least significant different (LSD) test. The SPSS 15.0 software package (SPSS, Chicago, IL, USA) was used for data analysis. *p* < 0.05 was considered significant.

## Supplementary Materials

Supplementary materials can be found at www.mdpi.com/1422-0067/17/10/1611/s1. References [41,42] are cited in the supplementary materials.

## References

[1] Hu J., Fan D., Lin X., Wu X., He X., He X., Wu X., Lan P. (2015). Safety and efficacy of sodium hyaluronate gel and chitosan in preventing postoperative peristomal adhesions after defunctioning enterostomy: A prospective randomized controlled trials. Medicine.

[2] Yang B., Gong C., Zhao X., Zhou S., Li Z., Qi X., Zhong Q., Luo F., Qian Z. (2012). Preventing postoperative abdominal adhesions in a rat model with PEG-PCL-PEG hydrogel. Int. J. Nanomed..

[3] Yuan F., Lin L.X., Zhang H.H., Huang D., Sun Y.L. (2016). Effect of carbodiimide-derivatized hyaluronic acid gelatin on preventing postsurgical intra-abdominal adhesion formation and promoting healing in a rat model. J. Biomed. Mater. Res. A.

[4] Ten Broek R.P., Issa Y., van Santbrink E.J., Bouvy N.D., Kruitwagen R.F., Jeekel J., Bakkum E.A., Rovers M.M., van Goor H. (2013). Burden of adhesions in abdominal and pelvic surgery: Systematic review and met-analysis. BMJ.

[5] Miller G., Boman J., Shrier I., Gordon P.H. (2000). Natural history of patients with adhesive small bowel obstruction. Br. J. Surg..

[6] Alpay Z., Saed G.M., Diamond M.P. (2008). Postoperative adhesions: From formation to prevention. Semin. Reprod. Med..

[7] Becker J.M., Stucchi A.F. (2004). Intra-abdominal adhesion prevention: Are we getting any closer. Ann. Surg..

[8] Beyene R.T., Kavalukas S.L., Barbul A. (2015). Intra-abdominal adhesions: Anatomy, physiology, pathophysiology, and treatment. Curr. Probl. Surg..

[9] Liu H.J., Wu C.T., Duan H.F., Wu B., Lu Z.Z., Wang L. (2006). Adenoviral-mediated gene expression of hepatocyte growth factor prevents postoperative peritoneal adhesion in a rat model. Surgery.

[10] Uguralp S., Akin M., Karabulut A.B., Harma B., Kiziltay A., Kiran T.R., Hasirci N. (2008). Reduction of peritoneal adhesions by sustained and local administration of epidermal growth factor. Pediatr. Surg. Int..

[11] Yen T.T., Thao D.T., Thuoc T.L. (2014). An overview on keratinocyte growth factor: From the molecular properties to clinical applications. Protein Pept. Lett..

[12] Wang X., Yu M., Zhu W., Bao T., Zhu L., Zhao W., Zhao F., Wang H. (2013). Adenovirus-mediated expression of keratinocyte growth factor promotes secondary flap necrotic wound healing in an extended animal model. Aesthet. Plast. Surg..

[13] Lopes J.B., Dallan L.A., Campana-filho S.P., Lisboa L.A., Gutierrez P.S., Moreira L.F., Oliveira S.A., Stolf N.A. (2009). Keratinocyte growth factor: A new mesothelial targeted therapy to reduce postoperative pericardial adhesions. Eur. J. Cardiothorac. Surg..

[14] Shen J., Xu Z.W. (2014). Combined application of acellular bovine pericardium and hyaluronic acid in prevention of postoperative pericardial adhesion. Artif. Organs.

[15] Ward B.C., Panitch A. (2011). Abdominal adhesions: Current and novel therapies. J. Surg. Res..

[16] Wei G., Chen X., Wang G., Jia P., Xu Q., Ping G., Wang K., Li X. (2015). Inhibition of cyclooxygenase-2 prevents intra-abdominal adhesions by decreasing activity of peritoneal fibroblasts. Drug Des. Dev. Ther..

[17] Wei G., Chen X., Wang G., Fan L., Wang K., Li X. (2016). Effect of resveratrol on the prevention of intra-abdominal adhesion formation in a rat model. Cell. Physiol. Biochem..

[18] Robb W.B., Mariette C. (2014). Strategies in the prevention of the formation of postoperative adhesions in digestive surgery: A systematic review of the literature. Dis. Colon Rectum.

[19] Fayez J.A., Schneider P.J. (1987). Prevention of pelvic adhesion formation by different modalities of treatment. Am. J. Obstet. Gynecol..

[20] Yung S., Chan T.M. (2007). Mesothelial cells. Perit. Dial. Int..

[21] Mutsaers S.E. (2002). Mesothelial cells: Their structure, function and role in serosal repair. Respirology.

[22] Haney A.F., Doty E. (1994). The formation of coalescing peritoneal adhesions requires injury to both contacting peritoneal surfaces. Fertil. Steril..

[23] Fletcher N.M., Awonuga A.O., Abusamaan M.S., Saed M.G., Diamond M.P., Saed G.M. (2016). Adhesion phenotype manifests an altered metabolic profile favoring glycolysis. Fertil. Steril..

[24] Saed G.M., Fletcher N.M., Diamond M.P. (2016). The creation of a model for ex vivo development of postoperative adhesions. Reprod. Sci..

[25] Saed G.M., Kruger M., Diamond M.P. (2004). Expression of transforming growth factor-β and extracellular matrix by human peritoneal mesothelial cells and by fibroblasts from normal peritoneum and adhesions: Effect of Tisseel. Wound Repair Regen..

[26] Chegini N. (2002). Peritoneal molecular environment, adhesion formation and clinical implication. Front. Biosci..

[27] Kawanishi K., Nitta K. (2015). Cell sheet-based tissue engineering for mesothelial cell injury. Contrib. Nephrol..

[28] Bertram P., Tietze L., Hoopmann M., Treutner K.H., Mittermayer C., Schumpelick V. (1999). Intraperitoneal transplantation of isologous mesothelial cells for prevention of adhesions. Eur. J. Surg..

[29] Guo Q., Li Q.F., Liu H.J., Li R., Wu C.T., Wang L.S. (2008). Sphingosine kinase 1 gene transfer reduces postoperative peritoneal adhesion in an experimental model. Br. J. Surg..

[30] Finch P.W., Rubin J.S. (2006). Keratinocyte growth factor expression and activity in cancer: Implications for use in patients with solid tumors. J. Natl. Cancer Inst..

[31] Kovacs D., Raffa S., Flori E., Aspite N., Briganti S., Cardinali G., Torrisi M.R., Picardo M. (2009). Keratinocyte growth factor down-regulates intracellular ROS production induced by UVB. J. Dermatol. Sci..

[32] Abo T., Nagayasu T., Hishikawa Y., Tagawa T., Nanashima A., Yamayoshi T., Matsumoto K., An S., Koji T. (2010). Expression of keratinocyte growth factor and its receptor in rat tracheal cartilage: Possible involvement in wound healing of the damaged cartilage. Acta Histochem. Cytochem..

[33] Belleudi F., Scrofani C., Torrisi M.R., Mancini P. (2011). Polarized endocytosis of the keratinocyte growth factor receptor in migrating cells: Role of SRC-signaling and cortactin. PLoS ONE.

[34] Cheong Y.C., Laird S.M., Li T.C., Shelton J.B., Ledger W.L., Cooke I.D. (2001). Peritoneal healing and adhesion formation/reformation. Hum. Reprod. Update.

[35] Krüger-Szabó A., Aigner Z., Balogh E., Sebe I., Zelkó R., Antal I. (2015). Microstructural analysis of the fast gelling freeze-dried sodium hyaluronate. J. Pharm. Biomed. Anal..

[36] Yates A.C., Stewart A.A., Byron C.R., Pondenis H.C., Kaufmann K.M., Constable P.D. (2006). Effects of sodium hyaluronate and methylprednisolone acetate on proteoglycan metabolism in equine articular chondrocytes treated with interleukin-1. Am. J. Vet. Res..

[37] Rotolo S., Ceccarelli S., Romano F., Frati L., Marchese C., Angeloni A. (2008). Silencing of keratinocyte growth factor receptor restores 5-fluorouracil and tamoxifen efficacy on responsive cancer cells. PLoS ONE.

[38] Oelmann E., Haghgu S., Kulimova E., Mesters R.M., Kienast J., Herbst H., Schmitmann C., Kolkmeyer A., Serve H., Berdel W.E. (2004). Influence of keratinocyte growth factor on clonal growth of epithelial tumor cells, lymphoma and leukemia cells and on sensitivity of tumor cells towards 5-fluorouracil in vitro. Int. J. Oncol..

[39] Kanuga S. (2013). Cryotherapy and keratinocyte growth factor may be beneficial in preventing oral mucositis in patients with cancer, and sucralfate is effective in reducing its severity. J. Am. Dent. Assoc..

[40] Peyton C.C., Keys T., Tomblyn S., Burmeister D., Beumer J.H., Holleran J.L., Sirintrapun J., Washburn S., Hodges S.J. (2012). Halofuginone infused keratin hydrogel attenuates adhesions in a rodent cecal abrasion model. J. Surg. Res..

[41] Nair S.K., Bhat I.K., Aurora A.L. (1974). Role of proteolytic enzyme in the prevention of postoperative intraperitoneal adhesions. Arch. Surg..

[42] Leach R.E., Burns J.W., Dawe E.J., SmithBarbour M.D., Diamond M.P. (1998). Reduction of postsurgical adhesion formation in the rabbit uterine horn model with use of hyaluronate/carboxymethylcellulose gel. Fertil. Steril..

[43] Mahdy T., Mohamed G., Elhawary A. (2008). Effect of methylene blue on intra-abdominal adhesion formation in rats. Int. J. Surg..

[44] Lei J., Ma J., Ma Q., Li X., Liu H., Xu Q., Duan W., Sun Q., Xu J., Wu Z. (2013). Hedgehog signaling regulates hypoxia induced epithelial to mesenchymal transition and invasion in pancreatic cancer cells via a ligand-independent manner. Mol. Cancer.

[45] Schmittgen T.D., Livak K.J. (2008). Analyzing real-time PCR data by the comparative C(T) method. Nat. Protoc..

