# Supplementary material for: Keratinocyte Growth Factor Combined with a Sodium Hyaluronate Gel Inhibits Postoperative Intra-Abdominal Adhesions
Source: Int J Mol Sci. 2016 Sep 22;17(10):1611. doi: 10.3390/ijms17101611 (PMC5085644; doi:10.3390/ijms17101611)
Supplement: Supplementary file 1 [file ijms-17-01611-s001.pdf]

Supplementary Material: KGF Combined with a Sodium Hyaluronate Gel Inhibits Postoperative Intra-Abdominal Adhesions

Guangbing Wei, Cancan Zhou, Guanghui Wang, Lin Fan, Kang Wang and Xuqi Li

Table S1. The numerical scoring of the adhesions described by Nair, S.K. et al. [41].

Grade	Criteria
0	No adhesion band is present.
1	A single adhesion band forms between the viscera or between a viscus and the abdominal wall.
2	Two bands form between the viscera or between the viscera and the abdominal wall.
3	More than two bands form between the viscera or between the viscera and the abdominal wall, or the whole intestine forms a mass without adhering to the abdominal wall.
4	The viscera have directly adhered to the abdominal wall irrespective of the number of bands.

Table S2. The numerical scoring of adhesions described by Leach, R.E. et al. [42].

Grade	Criteria
0	No adhesion band is present.
1	An avascular band with a filmy thickness is formed.
2	A moderately vascularized band(s) with medium thickness is formed.
3	A well vascularized band(s) with dense thickness is formed.

Table S3. The numerical scoring of inflammation based on histology.

Grade	Inflammation
0	Nil.
1	Giant cells, occasional scattered lymphocytes and plasma cells.
2	Giant cells with increased numbers of admixed lymphocytes, plasma cells, eosinophils, and neutrophils.
3	Many admixed inflammatory cells; micro abscesses are present.

Table S4. Primers for real-time RT-PCR.

Genes	Primer Sequences
TGF-β1	P1: 5′-CATTGCTGTCCCGTGCAGA-3′<br>P2: 5′-AGGTAACGCCAGGAATTGTTGCTA-3′
Fibrinogen	P1: 5′-GGCAGATACTACTGGGGTGG-3′<br>P2: 5′-ATGCTTGGGGGACTATTGCTG-3′
α-SMA	P1: 5′-CCGGGAGAAAATGACTCAAA-3′<br>P2: 5′-GCGTCCAGAGGCATAGAGAG-3′
GAPDH	P1: 5′-ACCACAGTCCATGCCATCAC-3′<br>P2: 5′-TCCACCACCCTGTTGCTGTA-3′
